# Hyperuricemia and Its Association With Ischemic Stroke

**DOI:** 10.7759/cureus.18172

**Published:** 2021-09-21

**Authors:** Jaskamal Padda, Khizer Khalid, Sandeep Padda, Nymisha L Boddeti, Bhavsimran S Malhi, Rohini Nepal, Ayden Charlene Cooper, Gutteridge Jean-Charles

**Affiliations:** 1 Internal Medicine, Jersey City (JC) Medical Center, Orlando, USA; 2 Internal Medicine, Advent Health & Orlando Health Hospital, Orlando, USA

**Keywords:** ischemic stroke, hyperuricemia, urate lowering agents, serum uric acid, atherosclerotic, hypertension

## Abstract

Elevated serum uric acid (SUA) levels have been associated with an increased risk of cardiovascular (CV) disease and acute ischemic stroke (AIS) as well as many other medical conditions. AIS is a CV complication that is the second most common cause of mortality worldwide. It results from reduced blood flow to the brain by means of thrombosis, embolism, or systemic hypoperfusion. Studies have demonstrated an association between SUA levels and CV events, with a significant dose-response relationship between elevated SUA levels and stroke risk. Since the relationship between SUA levels and AIS risk has been established, studies are also being conducted in order to evaluate whether antihyperuricemic drugs can lower this risk. Allopurinol use in hyperuricemic patients has been shown to decrease the risk of major CV events, which include AIS. This narrative review aims to investigate the role of SUA as an independent risk factor for AIS along with the proposed biological mechanisms by thoroughly appraising research findings from relevant full-text articles and abstracts indexed in PubMed and the Cochrane Library. In this literature, we will be discussing hyperuricemia, AIS, the association between the two, and the use of antihyperuricemic medications on stroke prognosis. This review will also shed new light on studies that have begun to provide insight into the predictive role of hyperuricemia in AIS.

## Introduction and background

Stroke is the second most common cause of mortality in the world. It is responsible for 11% of deaths and is a major cause of disability worldwide [1]. It is the fifth leading cause of mortality in the United States, accounting for 17% of deaths [2]. As per the global burden of disease estimates, in 2016, there were 9.5 million new cases and 2.7 million deaths, related to people suffering from ischemic stroke. Also, 50% of healthy life lost due to complications of ischemic stroke targets people under the age of 70 years [3]. Stroke is an enormous public health issue, with 77% of strokes being first-time events and 87% of all strokes being ischemic in nature [4]. Ischemic stroke is defined as the loss of neurologic function due to the sudden cessation of blood circulation to the brain [5]. It can be the result of multiple occurrences such as the blockage of vessels within the brain, atherosclerosis which affects cerebral circulation, and embolism. These causations result in cerebral inflammation that eventually provokes cell death [6]. Thus, the prevention of ischemic stroke is critical to reducing the burden of the disease.

Hyperuricemia refers to the elevation of uric acid in the plasma. It has no widely accepted single cut-off value, however, values above 6.8mg/dL are generally considered elevated in adults [7]. Hyperuricemia has been shown to have a positive association with CV morbidity and mortality [8,9]. In the Rotterdam Cohort study of 4,385 participants, uric acid was found to be a strong risk factor for stroke [9]. However, a study conducted by Chamorro et al. concluded good clinical outcomes in patients with acute ischemic stroke (AIS) and increasing uric acid levels [10]. Furthermore, there are many more studies that indicated worsening outcomes in AIS patients with the rise in serum uric acid (SUA) levels [11,12]. This seemingly inconsistent and conflicting data necessitates more research. This review aims to study the association between hyperuricemia and stroke and the role of anti-hyperuricemic medication on stroke prognosis.

## Review

Hyperuricemia

Uric acid is the final product of protein and purine metabolism, which is a poorly soluble component and is mostly excreted out of the body via the urine [13]. The cut-off values differ between each gender, normal uric acid levels in males and females are 3.4-7.0mg/dL and 2.4-6.0mg/dL, respectively [14,15]. According to the National Health and Nutrition Examination Survey (NHANES), the incidence and prevalence of hyperuricemia remained stable in the United States from 2007 to 2016 and the prevalence rate from 2015 to 2016 was 20.2% in men and 20.0% in women [16]. Hyperuricemia is associated with an increased risk of CV disease and AIS, along with kidney disease, diabetes, non-alcoholic fatty liver disease, and obesity [13,17]. Many mechanisms of CV disease have been suggested in hyperuricemia such as uric acid-induced endothelial dysfunction, platelet adhesiveness and aggregation, oxidative metabolism [14].

Uric acid is also known to have both prooxidant and antioxidant activity [17]. It has been known as an effective antioxidant and it efficiently scavenges the body for up to 60% of singlet oxygen molecules and free radicals. Under physiological conditions, uric acid’s effects can be as effective as ascorbate, which is another important antioxidant in plasma [18]. A study conducted by Ames et al. proved that at physiological concentrations, the functions of urate consist of reducing oxo-heme oxidant formed by a peroxide reaction with hemoglobin inhibits lipid peroxidation of erythrocyte (RBC) ghost cells, and protects RBCs from lysis by peroxidative damage [19]. In terms of being a prooxidant, one of the earliest effects of uric acid-exposed cells is to generate oxidative stress resulting in the production of reactive oxygen species (ROS). As a result, ROS have been tied to the development of local inflammation, impairment of nitric oxide generation, renin-angiotensin system activation, accumulation of fat, and insulin resistance. Therefore, this results in a cascade of events causing damage to various cell types including vascular endothelial and smooth muscle cells, renal tubular cells, adipocytes, hepatocytes [20].

Many transport proteins involved in the transport of uric acid are essential in the treatment of hyperuricemia. Solute carrier family 22 - member 12 (URAT1/SLC22A12), solute carrier family 2 - facilitated glucose transporter member 9 (GLUT9/SLC2A9), and ATP-binding cassette superfamily G member 2/breast cancer resistance protein (ABCG2/BCRP) are urate transporters which play a vital role in the regulation of uric acid levels. Among these transporters, ABCG2 exporter dysfunction is known to be a major and common cause of hyperuricemia since it acts on both the kidney and GI systems [13,21].

Treatment is based on whether patients are symptomatic or asymptomatic. Asymptomatic hyperuricemic patients do not require any urate-lowering therapy [13,14]. For these patients, it is adequate to make lifestyle changes such as an increase in exercise, dietary changes, and reduction in alcohol intake in order to reduce SUA levels. Symptomatic patients can present with gout, nephrolithiasis, uric acid renal disease, further requiring pharmacotherapeutic management. Effective management of hyperuricemia includes inhibiting synthesis and reabsorption of uric acid and increasing the excretion of uric acid. The main categories of drugs include xanthine oxidase inhibitors (reduces the production of uric acid), URAT1 inhibitors (increases the excretion of uric acid), uricase inhibitors (regulates the metabolic hydrolysis of uric acid), and non-purine analog agents [13].

Ischemic stroke

Ischemic stroke results from reduced blood flow to the brain by means of thrombosis, embolism, or systemic hypoperfusion. Thrombosis is usually preceded by atherosclerotic disease, arterial dissection, fibromyalgia, or inflammatory conditions causing vascular dysfunction [22-24]. Circulatory irregularities and a lack of blood flow set off a complicated chain of biochemical and molecular reactions that eventually result in ischemic cell death [23]. Neuronal death and irreversible loss of neuronal function are the end results of the ischemic cascade triggered by stroke. Due to low respiratory reserve and total reliance on aerobic metabolism, brain tissue is particularly vulnerable. The existence of collateral circulation brings about a spectrum of severity, which is commonly found in the afflicted area of the brain. Hence, some portion of brain parenchyma becomes subject to death whereas other portions tend to have a limited injury with a chance of recovery (penumbra) [24].

In AIS, the objective of treatment is to maintain tissue viability in places where perfusion is reduced but also remains sufficient to prevent infarction. The tissue in these areas of oligemia is protected by enhancing the collateral flow and re-establishing blood flow to the affected areas as swiftly as possible. Table 1 discusses the diagnostic criteria while Table 2 demonstrates the management of ischemic stroke [22,25].

**Table 1:** Prehospital/clinical diagnostic criteria for stroke This table discusses two methods to diagnose stroke in the prehospital and clinical settings.

6S Method	BEFAST Method
Sudden – sudden onset of symptoms	Balance – loss of balance/dizziness
Slurred Speech	Eyes – disturbance of vision (uni/bilateral)
Side Weak – face, arm, leg	Face – facial droop
Spinning – vertigo	Arm – weakness
Severe Headache	Speech Test - slurred
Seconds – note the time of symptom onset and rush to the hospital	

**Table 2:** Ischemic stroke: step-by-step assessment and treatment ABC, airway breathing circulation; ECG, electrocardiogram; CBC, complete blood count; PT, prothrombin time; INR, international normalized ratio; aPTT, activated partial thromboplastin time; CT scan, computerized tomography scan; MRI, magnetic resonance imaging; IV tPA, intravenous alteplase; DVT, deep vein thrombosis.

Management of ischemic stroke	
1. Clinical diagnosis of stroke	See Table 1
2. Medically stabilize patient	a. ABC
3. Evaluate for reversible causes of neurological symptoms	a. Fingerstick glucose
	b. ECG
	c. CBC
	d. Troponin
	e. PT and INR
	f. aPTT
	g. If taking oral anticoagulants – clotting time, thrombin time, factor Xa activity assay
4. Determine ischemic vs hemorrhagic	a. Head CT scan non-contrast
	i. If presenting <6hrs – CT angiogram of the head and neck
	ii. If presenting 6-24hrs with ischemic stroke diffusion weighted-MRI or MRI perfusion
5. Management of stroke	a. <3hrs presentation – IV tPA infusion (eligible patients can extend time to <4.5hrs)
	b. Endovascular therapy
	c. Optimization of blood pressure, temperature, blood glucose, and nutrition
	d. DVT prophylaxis
	e. Depression screening
	f. Management of cerebellar/cerebral edema
	g. Anti-epileptic treatment in patients who experience recurrent seizures
	h. Cardiac evaluation with antiplatelet and antithrombotic treatment
	i. Statins

The prognosis of ischemic stroke varies greatly among individuals. The severity of the stroke, the implicated structures, the time to treatment initiation, the affected area, the time to identification and diagnosis, duration and intensity of physical and occupational therapy, and baseline functionality all play a role in the prognosis [22].

Hyperuricemia and ischemic stroke

The association between hyperuricemia and AIS remains controversial despite two recent meta-analytical reviews of prospective studies demonstrating a significant relationship [14,26]. The largest of the component studies, the Atherosclerosis Risk in Communities (ARIC), found hyperuricemia to no longer be independently associated with ischemic stroke after adjusting for diuretic-treated hypertension [27]. However, a case-cohort study in 2020 [28] analyzed the relationship between hyperuricemia and ischemic stroke in a large community-based population that had been enrolled in the national Reasons for Geographic and Racial Differences in Stroke study (REGARDS) [29], while also quantifying the role of blood pressure and other confounders. The study not only confirmed hyperuricemia to be an independent risk factor for ischemic stroke but also showed substantial attenuation of the hyperuricemia-stroke association when adjusted for hypertension severity upon further mediation analyses. This suggests that severe hypertension may be a mediator of the pathway between hyperuricemia and stroke. The association between hypertension and hyperuricemia is further supported by epidemiological and animal studies [30-33] as well as clinical trials [34]. A meta-analysis in 2017 [35] also showed a significant dose-response relationship between elevated SUA levels and stroke risk, reporting an increase in stroke risk by about 10% for every 1 mg/dL increase in SUA levels. To overcome the limitations associated with multivariate analyses, a large prospective study in 2019 [36] looked at the relationship between hyperuricemia and stroke in Chinese elderly subjects who were solely hyperuricemic without any other CV comorbidities. It was found that asymptomatic hyperuricemia increased the risk of both ischemic and hemorrhagic strokes. The hyperuricemic subjects had a twofold increased risk of stroke within three years, demonstrating that asymptomatic hyperuricemia can be a valuable predictor of stroke.

SUA paradoxically exhibits both antioxidant and prooxidant properties, with a protective role in the plasma and a pathogenic role within the cell [37]. Elevated SUA is significantly linked with CV risk factors such as hypertension, abdominal obesity, hyperlipidemia, and insulin resistance [38,39]. Several epidemiological studies have shown a J-shaped association between SUA and CV events [40,41], indicating that both low (<5.6 mg/dL in men and <4.3 mg/dL in women) and high (>7.1 mg/dL in men and >5.5 mg/dL in women) SUA levels increase mortality from CV disease [40]. One proposed mechanism by which hyperuricemia causes CV risk is due to its direct role in the development of atherosclerosis. Human atherosclerotic plaques contain more urate crystals than normal arterial walls [42] and the phagocytosis of the deposited urate crystals by polymorphonuclear leukocytes triggers the release of a variety of inflammatory mediators. The resulting inflammation of the vascular walls may lead to intimal damage, platelet activation, and coagulation cascades [43]. SUA may also indirectly promote atherosclerosis by inducing vascular endothelial dysfunction [44], activating an important atherosclerosis chemokine called monocyte chemotactic protein-1 [45], and stimulating proliferation on vascular smooth muscle cells [46]. The main reason underlying the endothelial dysfunction is proposed to be oxidative stress secondary to reduced nitric oxide bioavailability [47] following SUA entry into endothelial cells. The uric acid subsequently initiates multiple proinflammatory reactions that result in the release of ROS and potentiate the atherosclerotic process [48,49].

Antihyperuricemic medications and stroke prognosis

Since hyperuricemia has been associated with increased CV disease, many studies have been conducted in order to evaluate whether antihyperuricemic drugs can lower this risk. A study conducted in Denmark on a cohort followed over 18 years, aimed to investigate CV outcomes while taking allopurinol in hyperuricemic patients. The study consisted of 65,971 hyperuricemic patients (urate level >6.0mg/dL) of which 7,127 patients were already being treated with allopurinol. The researchers concluded that allopurinol use was associated with decreased risk of major CV events which included stroke [50]. Recently, a large study by Singh and Yu found a decrease in the risk of ischemic stroke by 9% with allopurinol use and an even greater decrease (12%-21%) with a longer duration of use. The beneficial effects of allopurinol were noted after six months of use. It was established that the patients on allopurinol who experienced a stroke were most likely to be older, white, and possibly women [51].

A study was conducted by Taheraghdam et al. to assess whether a three-month regimen in patients with high SUA and AIS would have a positive outcome. The study included 70 patients with both AIS and elevated SUA levels who were divided into treatment and placebo groups. It was concluded that the administration of allopurinol in these patients might increase their functional status without decreasing the mortality rate and that it may be beneficial to administer allopurinol at the time of acute events [52].

In the CARES trial that assessed the CV safety of febuxostat and allopurinol in gout, non-fatal stroke was seen in 2.3% of patients treated with febuxostat as well as allopurinol. The all-cause and CV mortalities were considerably elevated with febuxostat when compared to allopurinol. Overall, febuxostat and allopurinol had similar rates of major CV events in patients who had gout and coexisting CV disease [53]. The variances in mortality were most apparent in patients with lower baseline urate levels. The percentage of patients with serum urate levels <5 mg/dL on week 2 was 1.8-fold higher with febuxostat, indicating a dose-response relationship between the amount of urate-lowering and mortality [54]. Excessive lowering of SUA levels is thus a potential danger that needs to be acknowledged while giving urate-lowering therapy. Although there were positive outcomes with the use of antihyperuricemic drugs, larger trials are required to solidify the use of these drugs in the treatment and prevention of AIS in hyperuricemic patients.

Literature research

Table 3 consists of studies conducted in an effort to relate hyperuricemia to ischemic stroke. A literature search of databases, such as PubMed, PubMed Central, and Google Scholar, was conducted in July 2021 for studies related to “hyperuricemia and ischemic stroke”. Ten studies have been included in this review. The following inclusion criteria were used based on the authors’ discretion: patients with confirmed ischemic stroke, patients with confirmed hyperuricemia, and sample size >50 [9,17,36,55-61].

**Table 3:** Hyperuricemia and its association with ischemic stroke SD, standard deviation; SUA, serum uric acid; N/A, not available; NIDDM, non-insulin-dependent diabetes mellitus; CHD, coronary heart disease; MI, myocardial infarction; AIS, Acute Ischemic Stroke

Referenced Trial	Sample Size	Study Participants	Mean Uric acid Levels	Primary Outcomes	Objective	Conclusion
Mehrpour et al. (2012) Tehran, Iran [55]	55	46 – Ischemic stroke 9 – Hemorrhagic stroke	5.94 mg/dL (SD 1.70)	26 (47.3%) patients had hyperuricemia	To determine the SUA levels in acute stroke patients	The prevalence of hyperuricemia in acute stroke patients was significantly higher than normal population
Milionis et al. (2005) Greece [56]	329	163 – Ischemic stroke 166 - controls	5.6 ± 1.7 mg/dL vs. 4.8 ± 1.4 mg/dL (stroke patients vs control respectively)	49 (30.1%) stroke patients had hyperuricemia	To assess the association between SUA and acute ischemic/nonembolic stroke	Elevated SUA was associated with increased risk for acute ischemic/nonembolic stroke in elderly patients
Takagi (1982) Japan [57]	314	314 men from village of Ushibuka (30 resulted in AIS)	6.9 ± 2.0 mg/dL vs. 5.8 ± 1.8 mg/dL (stroke vs no stroke respectively)	High SUA levels were found in patients who experienced a stroke	To clarify the risk factors for stroke from an epidemiological point of view	SUA levels showed a positive correlation to the incidence of AIS
Lehto et al. (1998) Finland [58]	1,017	551 men and 466 women with NIDDM (114 participants had a fatal/non-fatal stroke)	N/A	Elevated SUA level (>295 umol/L) is significantly associated with AIS	To investigate SUA as an independent risk factor of stroke in NIDDM patients without nephropathy	Hyperuricemia is a strong indicator of stroke events in NIDDM patients
Chien et al. (2005) Taiwan [59]	3,602	1,703 men and 1,899 women (155 participants resulted in AIS)	5.67 mg/dL (SD 1.68) vs 6.91 mg/dL (SD 2.95); baseline vs fifth followup (8 year) respectively	As uric acid increased one unit, men had 1.24 times and women had 1.60 times higher risk for stroke events	To investigate the association of SUA levels in CHD and AIS	Hyperuricemia is associated with increased stroke events
The Rotterdam Study (2006) Netherlands [9]	4,385	Out of total, 381 participants experienced AIS	Baseline is 309 umol/L with a hazard ratio of 1.77 (1.10-2.83) for ischemic stroke and 1.68 (0.68-4.15) for hemorrhagic stroke	High SUA levels were associated with the right of MI and AIS	To investigate the association between SUA and CHD and stroke	SUA is a strong risk factor for MI and stroke
The ARIC Study (2006) USA [60]	13,413	Out of total, 381 participants experienced AIS	5.97 ± 1.52 mg/dL with 24.1% of participant having uric acid >6.9 mg/dL	A positive moderate association between SUA and AIS was noted	To investigate the relation between SUA and AIS	SUA is an independent indicator of ischemic stroke among patients who are not using diuretics
Khalil et al. (2020) Bangladesh [61]	388	169 cases (AIS) and 169 controls	6.03 ± 1.84 mg/dL vs. 4.34 ± 1.60 mg/dL (cases vs control respectively	31% of cases had hyperuricemia; For every one-unit increase in SUA level, the rate of having an ischemic stroke was increased by 25%	To examine the relation between SUA level and ischemic stroke; also assessed gender-based differences	SUA is considerably associated with acute phase of ischemic stroke; gender-based analysis exhibits this in females only.
Irfan et al. (2020) Pakistan [17]	148	74 cases (AIS) and 74 controls	5.94 ± 1.70 mg/dL	48.6% of participants in the case group had hyperuricemia and ischemic stroke	To determine the connection between hyperuricemia and AIS	The prevalence of hyperuricemia in patients with AIS was higher compared to the healthy population
Tu et al. (2019) Shanghai [36]	3243	1784 males and 1459 females; 268 participants had a stroke	5.6 ± 1.6 mg/dL vs 8.3 ± 1.5 mg/dL (Baseline vs hyperuricemic)	Hyperuricemic subjects had a higher cumulative incidence of ischemic and hemorrhagic stroke	To evaluate whether asymptomatic hyperuricemia is associated with an increased risk of stroke in elderly subjects without comorbidities	Asymptomatic hyperuricemia without comorbidities had a higher (2.32 fold) risk of incident stroke

## Conclusions

It is evident that hyperuricemia shares a relationship with ischemic stroke. Even though uric acid has many protective physiologic properties, many studies have linked elevated SUA levels to an increased risk of atherosclerosis, CV disease, hypertension, and oxidative damage, all of which ultimately result in an increased possibility of AIS. In individuals who have experienced AIS, the coexistence of hyperuricemia is found to be substantially higher than that in the general population. It has since been hypothesized that hyperuricemia is able to cause endothelial dysfunction through oxidative stress, resulting in inflammation and the release of ROS, therefore causing subsequent atherosclerosis resulting in eventual ischemic stroke. The aforementioned three studies have established that treatment with antihyperuricemic drugs resulted in a decreased risk of stroke and mortality, while others did not. Further investigation is still required to establish a strong relationship between hyperuricemia and AIS, along with research to support urate-lowering therapy as a targeted treatment in lowering mortality in patients with hyperuricemia and ischemic stroke.
